# Design of New-Generation Usable Forms of Topical Haemostatic Agents Containing Chitosan

**DOI:** 10.3390/molecules22122240

**Published:** 2017-12-15

**Authors:** Dorota Zielińska, Marcin H. Struszczyk, Longina Madej-Kiełbik, Edyta Chmal-Fudali, Magdalena Kucharska, Maria Wiśniewska-Wrona, Kinga Brzoza-Malczewska

**Affiliations:** 1 R&D Project Department, Institute of Security Technologies MORATEX, Lodz 90-505, Poland; dzielinska@moratex.eu (D.Z.); lmadej@moratex.eu (L.M.-K.); efudali@moratex.eu (E.C.-F.); 2 Team of Biomaterials, Institute of Biopolymers and Chemical Fibres, Lodz 90-570, Poland; sekretarz_naukowy@ibwch.lodz.pl (M.K.); mwrona@ibwch.lodz.pl (M.W.-W.); biomater@ibwch.lodz.pl (K.B.-M.)

**Keywords:** chitosan, risk analysis, designing, haemostatic agents

## Abstract

Designing usable forms of topical haemostatic agents is the most important activity during the design process, resulting in strengthened functional properties of the final medical devices. This study aimed to propose indications for a research programme based on risk management supporting the development of two usable forms of a topical haemostatic agent: chitosan/alginate lyophilized foam and chitosan/alginate impregnated gauze. Both of the usable forms of the topical haemostatic agent, being the main part of the modified combat gauze, were fabricated using the chitosan/alginate complex. Risk analysis is helpful in developing an appropriate research programme, significantly reducing the risk to an acceptable level.

## 1. Introduction

The risk management process is of great importance when designing medical devices. It requires a strict determination of the potential hazards and the control tools as verification tests for the reduction of a risk to an acceptable level. The verification tests can be the main part of the optimal research programme.

A new-generation topical haemostatic agent is expected to satisfy the demands for adequate haemostatic capacities in injuries and surgical wounds, antibacterial activity to prevent primary and secondary infections, and safety in use. There are many topical haemostatic agent materials available on the medical market containing natural polymers, designed for curing wounds throughout the entire healing process. The most popular are haemostatic dressings that are prepared based on chitosan and alginate [1,2,3]. The materials benefit from their high absorption and haemostatic capacity due to the presence of calcium ions, which when released from alginate to the wound, activate platelets and accelerate haemostasis.

Another group of haemostatic dressing materials is based on chitin derivatives, such as chitosan. This group includes commercial dressings such as HemCon^®^ (Tricol Biomedical, Inc., Portland, OR, USA), RDH (Marine Polymer Technologies, Danvers, MA, USA), Syvek^®^ Patch (Marine Polymer Technologies, Danvers, MA, USA), Clo-Sur P.A.D. (Merit Medical Systems Inc., South Jordan, UT, USA), ChitoSeal^®^ (Luna Inc., Charlottesville, VA, USA), Traumastat (Ore-Medix, Salem, OR, USA), and Excel Arrest (Hemostasis LLC Co., Saint Paul, MN, USA) [4,5,6].

HemCon^®^ exhibits haemostatic action by direct adhesion to the wound, thus, accelerating the incorporation of erythrocytes to the growing clot. Clo-Sur PAD, Syvek^®^ Patch, and ChitoSeal^®^ are available in the form of haemostatic patches, with chitosan as the active substance. There is also a first aid dressing Tromboguard^®^, produced by TRICOMED S.A. in Lodz [7,8]. Moreover, the Sam Medical Company (Wilsonville, OR, USA) offers a chitosan haemostatic dressing, called Celox^®^ [9,10]. It is available in granulated form or as an applicator. Previous studies also report a dressing in the form of foam that is applied using a pressurized container. This dressing is being developed by the company Remedium Inc. (College Park, MD, USA), and is presently at the preclinical and clinical testing stage [11].

Two documents are helpful for the appropriate risk management process realization: EN ISO 14971:2012 [12] Standard, and, if a medical device contains animal tissue derived substance, EN ISO 22442-1:2007 Standard [13]. The realization of the risk analysis, being the main area of the risk management, allows for the identification of a vast area of the potential unconformities concerning the performance and safety of the analysed medical device.

The design process of a medical device, including the topical haemostatic agent, requires several verifications and validation stages, the effectiveness of which results from an appropriate methodology test and research programme composition. The application of the new forms of the chitosan/alginate micro- and nano-fibrids formulation, being the effect of the design process supported by a risk analysis process, will result in the improvement of the absorption capacity of amorphic constituents and increase in the internal surface that synergistically activates the haemostatic pathway. Moreover, the directional design of the usable forms of the topical haemostatic agents allows for the maximalization of the performance of the medical devices due to the localization and contact aspects. The synergistic effect resulting from the formulation of the haemostatic agent screening and the optimal selection of the haemostatic factor carriers constitutes product innovation (new topical haemostatic agent formulation) supported by process innovation (risk management process for the selection most proper performance).

The topical haemostatic agents are medical devices that are used mostly in case of an intense haemorrhage [5,14,15,16,17]. Several usable forms are applied in practice, such as impregnated gauze, tampon, foam, and powders [18].

The main aspects of the performance and safety of the products mentioned above should ensure the stable and strictly defined functionalities during the entire lifecycle.

The application of risk analysis supports the selection of an appropriate and safe research programme in order to:

- verify the performance (each usable feature and global, whole functionalities);
- verify the stability during storage and transportation; and,
- estimate the life-span of the products.

The risk analysis strictly supports the appropriate estimation of the verification tests that significantly reduce the risk to an acceptable level.

This study aimed to propose the guide for the estimation (appropriate for the design process) of a research programme based on the risk management process supporting the works on designing two usable forms of the topical haemostatic agent, including chitosan/alginate lyophilised foam and chitosan/alginate impregnated gauze.

Both usable forms of the topical haemostatic agent, being the main part of the modified combat gauze, were fabricated using the chitosan/alginate compositions. Moreover, the comparison of the main properties of clinically used topical haemostatic agents with the usable forms being developed was performed for the validation of the evaluated risk levels.

The present research was focused on two innovation areas:

(i) research design adaption including adaption of the risk management as an element of the design thinking for optimal identification of the verification tests as well as screening the optimal design pathway taking into account the interconnection of the performance features with the optimal verification method; and,
(ii) elaboration of the useful forms of the haemostatic topical agents based on the newly-developed chitosan-alginate fibrids complex taking into the account the future clinical application and defined performance of the advanced medical devices. Additionally, the effectivity of the risk management process was presented in aspect of the correlation with assumed performance, verification testing methods application conforming the suitability of the developed useful forms of the haemostatic topical agents.

## 2. Results and Discussion

### 2.1. Risk Analysis in the Area of the Main Functionalities of the Usable Forms of Topical Haemostatic Agents

**Figure 1:**
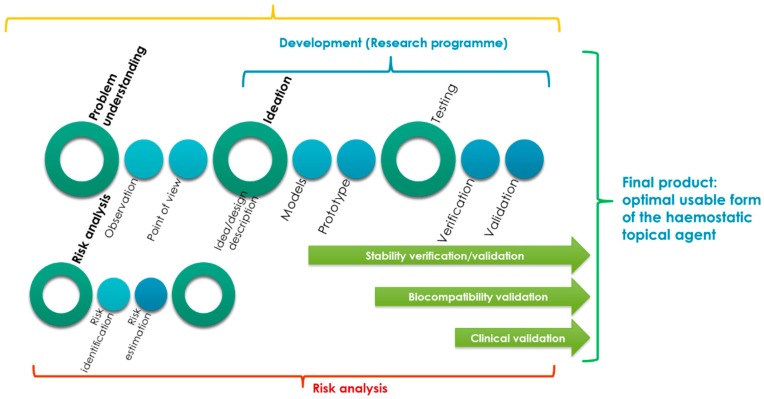
Proposal of the combination of design consideration and risk analysis processes for the development of the advanced medical devices—usable forms of topical haemostatic agents.

Figure 1 shows the combination of the design, design consideration and risk analysis processes proposed for the optimal design of usable forms of a topical haemostatic agent (powder, impregnated gauze, and foam).

The development of the usable form of the topical haemostatic agent was significantly supported by “understanding the problem to be solved” (as a main part of the design consideration process) and risk analysis (risk identification).

The processes mentioned above were helpful in describing the concept to be developed by the ideation of the models and prototypes of the final products that would be verified and validated during the testing phase.

The idea of creating the design of a usable form of the topical haemostatic agent included three areas: initialization activities, main design process, and the post-design processes.

The initialization process that were related to the identification of the problem is to be solved by an interdisciplinary team mostly by deeply empathizing with the end-users (practitioners and patients). At this stage, the initial assumptions for the raw materials were elaborated, and the technology (to be developed) and design of the final product were defined.

Moreover, the research programme was prepared based on the risk analysis results. The main document supporting the process of developing advanced medical devices, such as a usable form of a topical haemostatic agent, is the research programme that includes control tools, such as testing methods for risk reduction to an acceptable level.

During the main development activities, the models and prototypes of the usable forms of the topical haemostatic agents were verified and validated. The primary verification activities were focused on testing the usable properties, which define the functionalities of the medical devices.

The verification of the stability of assumed functionalities during storage to determine the lifespan of the designed medical devices was improved during the validation phase. It covered the biocompatibility testing (acc. to the guide of EN ISO 10993-1:2009 Standard [19]) of the selected optimal, usable form of the topical haemostatic agent. Subsequently, the main validation stage was implemented to indicate the reproductivity of the production process, as well as safety and performance, during the clinical study.

**Figure 2:**
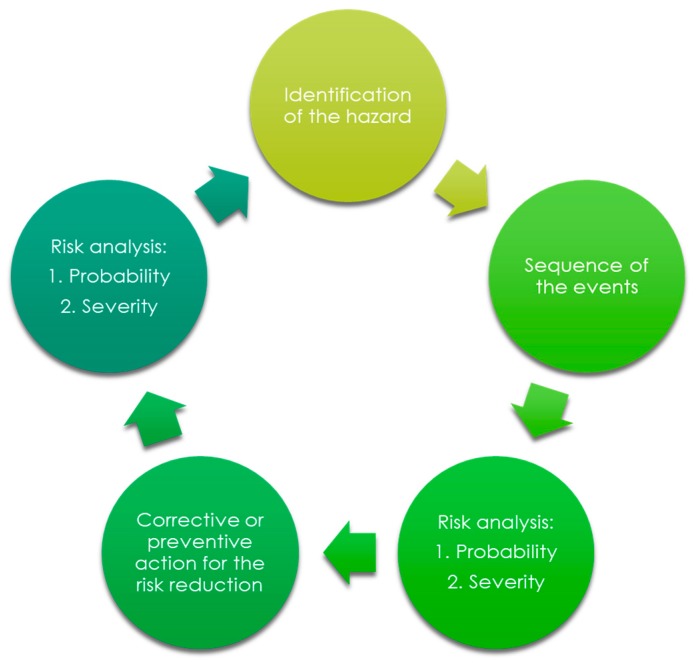
Risk analysis in designing the medical device—usable form of a haemostatic agent.

The process of risk analysis prepared in the aspect of the final medical device design is shown in Figure 2.

During the process of risk analysis, the following hazards were identified and estimated:

- biological hazards;
- environmental hazards;
- hazards originated from the leachable substances;
- hazards related to the non-proper use;
- hazards related to the production, quality control, and ageing;
- hazards related to the non-proper functionalities; and,
- other (such as clinical adverse events).

The design process (in the aspect of the selection) of the appropriate usable form of the haemostatic agent was strictly connected to the hazards related to the non-proper functionalities. The most important risk control tools for performance were evaluated directly indicating the assumed performance of the topical haemostatic agent, which finally influenced the usable form of the medical device.

The main verification tests to confirm the acceptable risk level in non-proper functionalities were as follows:

- surface density;
- moisture vapour transmission rate;
- absorption under free soaking;
- topography; and,
- chemical structure.

Moreover, the validation of the abovementioned properties is required for the estimation of the stability of the assumed functionalities during the simulated storage. Accelerated ageing is the main tool for the determination of the acceptance of observed changes in performance and identification of the reasons for the changes affecting the performance, as well as the safety of the newly developed medical device.

### 2.2. Analysis of the Stability of Main Functionalities of the Usable Forms of the Topical Haemostatic Agents

**Figure 3:**
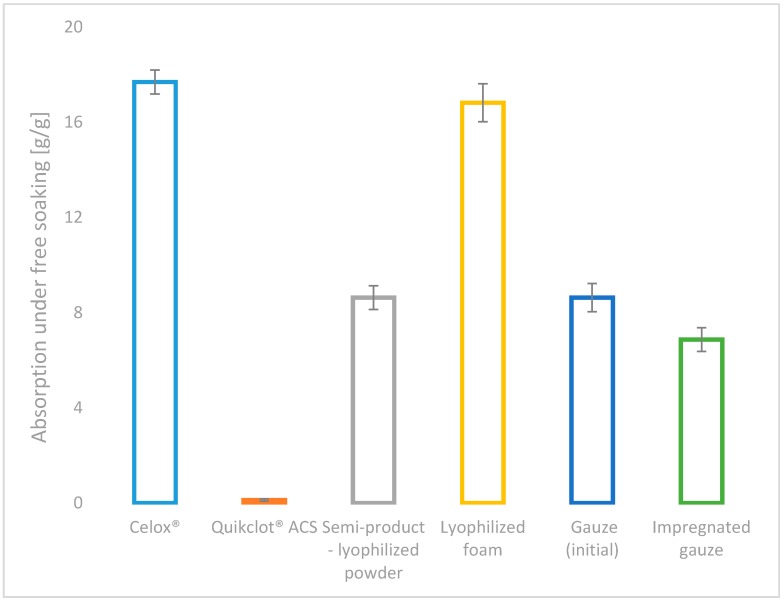
Absorption under free soaking of the references: Celox^®^, Quikclot^®^ ACS, semi-product—lyophilized powder [15] or gauze and the studied usable forms of the haemostatic agents: lyophilized foam and impregnated gauze.

**Figure 4:**
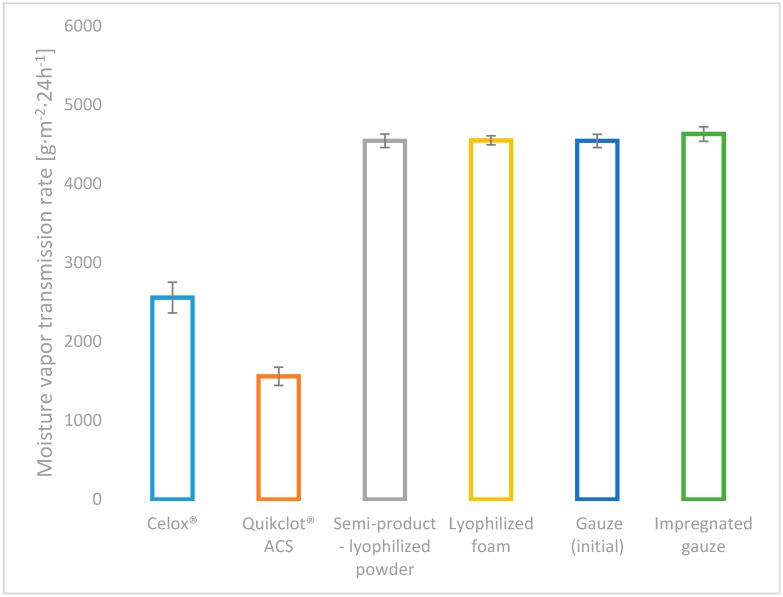
Moisture Vapour Transmission Rate (MVTR) of the references: Celox^®^, Quikclot^®^ ACS, semi-product—lyophilized powder [15], gauze and the studied usable forms of the haemostatic agents: lyophilized foam and impregnated gauze.

All of the developed variants of haemostatic agents (powder: chitosan/alginateCa/Na complex, lyophilized foam: complex chitosan/alginateCa/Na and impregnated gauze: chitosan/alginateCa/Na complex), as well as the commercial topical haemostatic agents (Celox^®^—haemostatic granules and Quikclot^®^ ACS) were initially characterized by the absorption under free soaking and moisture vapour transmission rate (MVTR), as shown in Figure 3 and Figure 4.

The value of the absorption under free soaking supports the estimation of the affinity of the topical haemostatic agents for the initial absorption of the amorphic constituents of the blood, and it indirectly affects the initiation of blood clotting by increasing the concentration of natural clotting factors.

Among the studied usable forms of the topical haemostatic agents, the lyophilized foam showed the value of absorption under free soaking when compared with Celox^®^ commercial product, promoting it for application in massive haemorrhage, internal application and filling deep injuries that are connected with massive blood loss. The impregnation of gauze with the haemostatic agents yielded the reduction of absorption under free soaking by approximately 20% (when compared to values detected for initial gauze and the semi-product—lyophilized powder), resulting from the coating of the gauze fibres by the haemostatic agent particles. The value of the studied parameter was lowered by 60% as compared with the foam-usable form. The lowest absorption under free soaking for Quikclot^®^ ACS was found, indicating the absence of the susceptibility to the concentration of non-morphotic fluids of blood under the test conditions.

High values of absorption under free soaking reduce the risk of uncontrolled bleeding and promote the acceptance of the clinical application of the studied usable forms in a wide range of the applications, such as surgical interventions, medical first aid for massive bleeding, haemostasis emergency, bleeding resulting from multiple defects, combating injuries, and in patients with haemostasis problems.

All of the developed usable form variants of haemostatic agents amounted to higher MVTR values than commercial haemostatic agents, as shown in Figure 4 (by approx. 40% in relation to Celox^®^ and by approx. 65% to Quikclot^®^ ACS).

No differences were detected in the value of MVTR for the semi-products: the powder and gauze, as well as the usable forms. MVTR as a measure of breathability has contributed to gas exchange in the wound environment, which influenced further wound healing.

Moreover, the stability testing of the assumed performance of the developed usable forms of topical haemostatic agents during storage and transportation is required to estimate the probability of risk of the non-compliant functionalities. The aspect mentioned above of the risk could be estimated during the accelerated ageing study.

**Figure 5:**
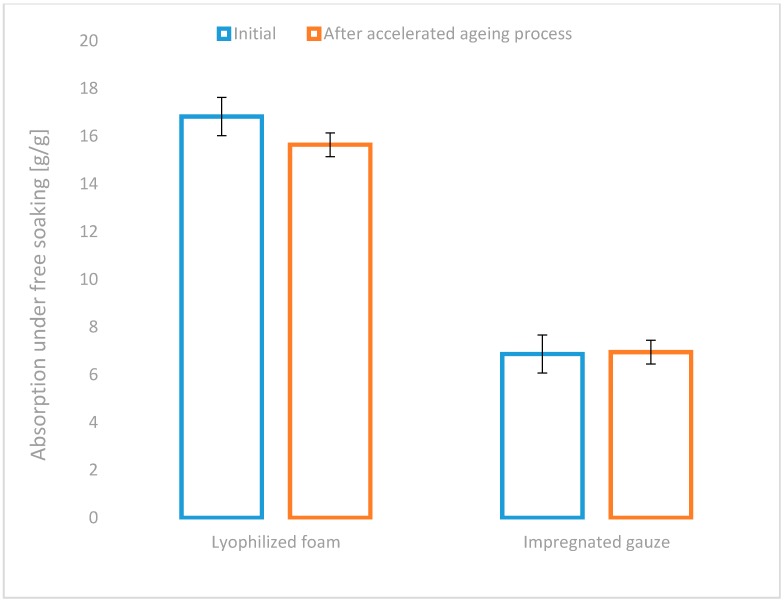
Absorption under free soaking of the studied usable forms of the haemostatic agents before and after accelerated ageing.

An insignificant decrease in absorption under free soaking in the lyophilized foam by approximately 7% after the accelerated ageing test was found (Figure 5). The absence of significant change in the above parameter in the second tested usable form was also detected. The results of the test confirm the stability of the tested parameters of newly developed usable forms of the haemostatic agents.

**Figure 6:**
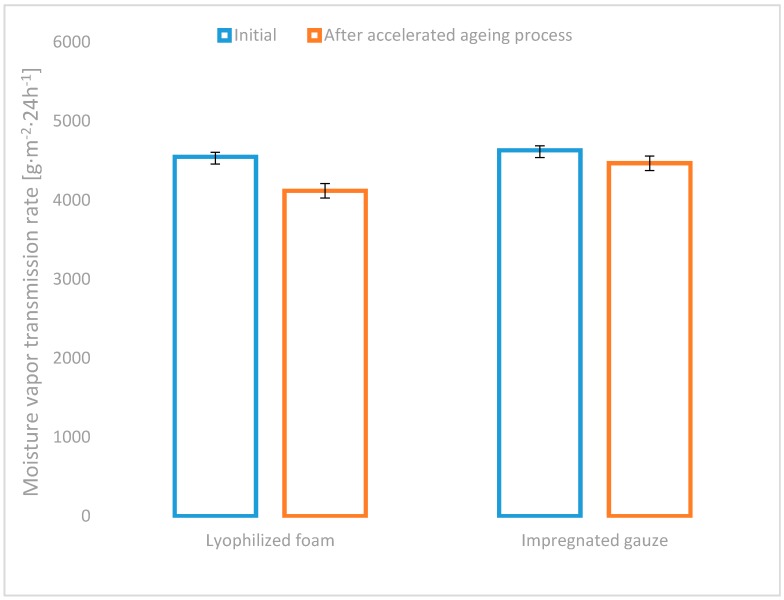
MVTR of the studied usable forms of the haemostatic agents before and after accelerated ageing.

A similar phenomenon was found when determining the MVTR values. The insignificant decrease of MVTR by approximately 9% was detected for the lyophilized foam, whereas the reduction in the impregnated gauze amounted to approximately 4% under accelerated ageing conditions (Figure 6).

The risk analysis that was performed for the detected parameters of the studied usable forms of the topical haemostatic agents indicated the acceptance of their performance stability. The complex analysis of usable parameters indicates that lyophilized foams are suitable for clinical use, mainly as haemostatic agents for the intraoperative surgical interventions, haemostasis emergency, bleeding resulting from multiple defects, and combat injuries, whereas the impregnated gauze as medical first aid in case of massive bleeding, haemostasis emergency, combat injuries, and in patients with haemostasis problems.

### 2.3. Structural Changes in Usable Forms of the Topical Haemostatic Agents—Infrared Spectroscopy

**Figure 7:**
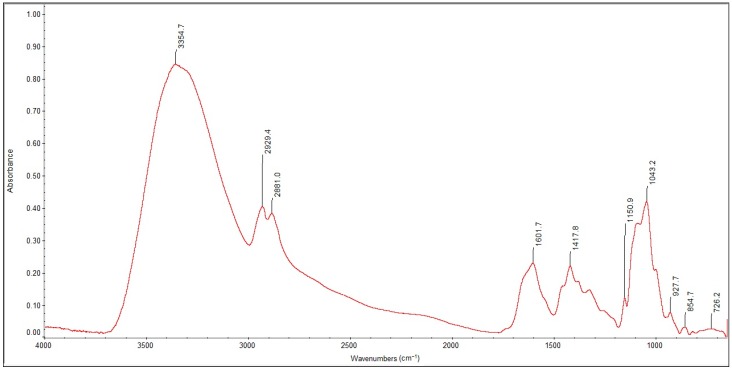
Fourier Transform Infrared Study (FTIR) spectra of the lyophilized foam before irradiation sterilization.

**Figure 8:**
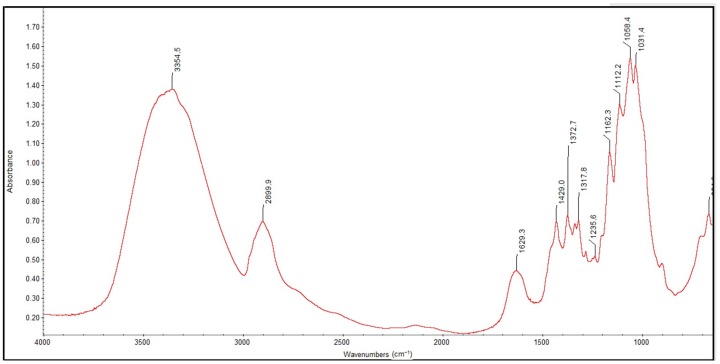
FTIR spectra of the impregnated gauze before irradiation sterilization.

Figure 7 and Figure 8 show the FTIR spectra in the 4000–400 cm^−1^ wavenumber range for the lyophilized foam and impregnated gauze before irradiation sterilization.

**Figure 9:**
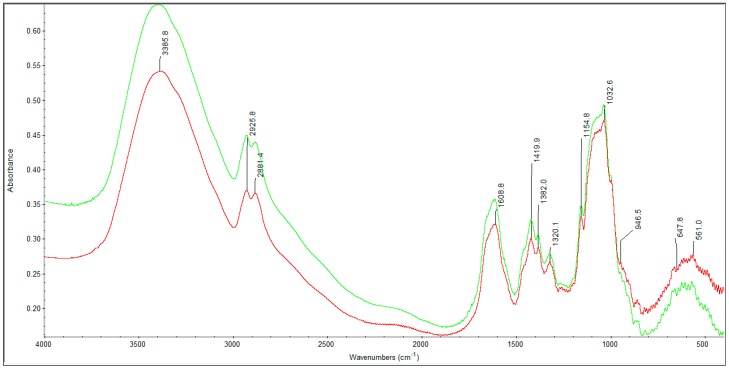
FTIR spectra of the sterilized lyophilized foam before and after the accelerated ageing process: − initial; − after one year of simulated ageing.

**Figure 10:**
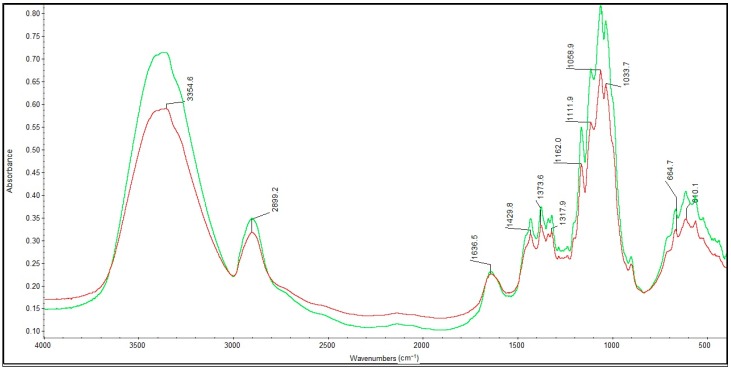
FTIR spectra of the sterilized impregnated gauze before and after the accelerated ageing: − initial; − after one year of simulated ageing.

Figure 9 and Figure 10 show the FTIR spectra in the 4000–400 cm^−1^ wavenumber range for the lyophilized foam and impregnated gauze before and after accelerated ageing.

The process of irradiation sterilization did not cause changes in the chemical structure of the sample. The dose of 25 kGy is safe. The FTIR spectra of lyophilized foam and impregnated gauze (before the accelerated ageing process) clearly indicate the observed absorption peaks corresponding to the chemical bond characteristics for chitosan. The main bands appearing in the FTIR spectrum were related to stretching vibrations of OH groups in the range of from 3750 cm^−1^ to 3000 cm^−1^ (λ = 3385 cm^−1^ and λ = 3354 cm^−1^), as deriving from the stretching vibration of the N-H and C-H bonds in –CH_2_ group (2925 cm^−1^ only for the lyophilized foam) and –CH_3_ (λ = 2881 cm^−1^ and λ = 2899 cm^−1^) [20].

Characteristic absorption bands λ = 1636 cm^−1^ and λ = 1608 cm^−1^ were derived from the absorption of the Amide II (bending vibrations in the N-H plane in amide groups). The bending vibrations of the methylene and methyl were also visible at λ = 1419 cm^−1^ and of λ = 1382 cm^−1^ (for the lyophilized foam) and λ = 1429 cm^−1^ and λ = 1373 cm^−1^ (for the impregnated gauze).

Absorption in the range of wavenumbers from 1160 cm^−1^ to 1000 cm^−1^ was attributed to vibrations in the CO group [21]. The bands that were located near λ = 1154 cm^−1^ and λ = 1162 cm^−1^ were related to the asymmetry of CO in the oxygen bridge resulting from the deacetylation of chitosan [20]. The bands near λ = 1080–1029 cm^−1^ were attributed to CO of the ring COH, COC and CH_2_OH. The small peaks λ = 870 cm^−1^ and λ = 899 cm^−1^ corresponded to the wagging of the saccharide structure of chitosan [22,23,24].

The separation of the FTIR spectra of the foams before and after accelerated ageing using PeakFit 4.12 (Systat Software Inc., San Jose, CA, USA) showed a shift in the absorption band at λ = 1596 cm^−1^ to a higher wavenumber (λ = 1589 cm^−1^) characterizing the deacetylated NH_2_ groups in the deacetylated chitosan and related to insignificant changes in the hydrogen bonding of the abovementioned groups. The absence of changes in the FTIR spectra of the impregnated gauze before and after accelerated ageing was detected after the applied separation of the FTIR spectra.

### 2.4. Topography of the Usable Forms of the Topical Haemostatic Agents

**Figure 11:**
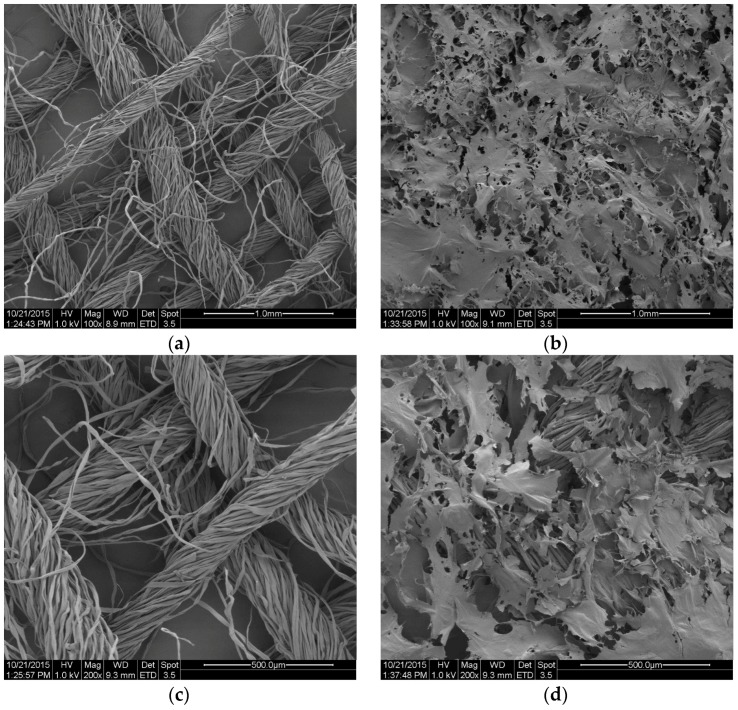
SEM microphotographs of: (**a**) gauze before impregnation (initial) at magnification 100×; (**b**) impregnated gauze at magnification 100×; (**c**) gauze before impregnation (initial at magnification 200×; and (**d**) impregnated gauze at magnification 200×.

**Figure 12:**
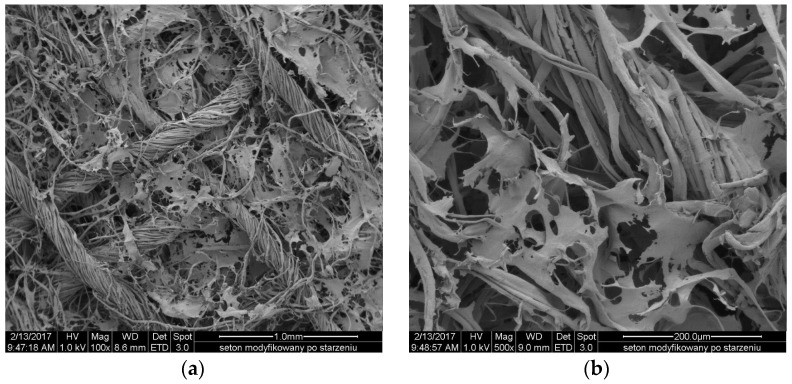
Scanning Electron Microscope Study (SEM) microphotographs of impregnated gauze after accelerated ageing: (**a**) at magnification 100×; and (**b**) at magnification 500×.

The Figure 11 and Figure 12 show the SEM microphotographs of the usable forms of the topical haemostatic agents: impregnated gauze and foams before and after accelerated ageing.

Figure 9 shows the SEM microphotographs of gauze before and after modification. The small-sized, empty spaces among the fibres were observed in SEM microphotograph of the initial sample, resulting from the woven structure of the gauze (Figure 11a,c).

After modification, all of the empty spaces between fibres became filled with chitosan/(calcium/sodium) alginate micro- and nano-fibrids. The process of impregnation yielded continuous distribution of the micro- and nano-fibrids onto the single filaments of yarns.

After the accelerated ageing process, no significant changes in the structure and topography of the impregnated gauze were observed. The structure of the impregnation was similar in view to those that were observed in the impregnated gauze before accelerated ageing.

**Figure 13:**
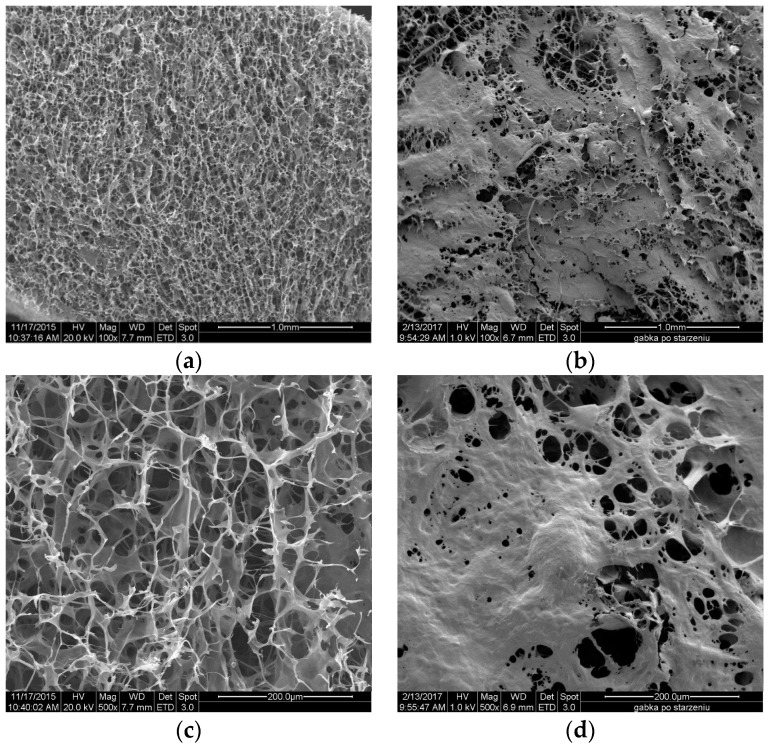
SEM microphotographs of (**a**) foam before accelerated ageing at magnification 100×; (**b**) foam after accelerated ageing at magnification 100×; (**c**) foam before accelerated ageing at magnification 500×; and (**d**) foam after accelerated ageing at magnification 500×.

Figure 13 shows the surface of lyophilized foam before and after accelerated ageing. The chitosan/(calcium/sodium) alginate micro- and nano-fibrids formed irregular spots after the accelerated ageing probably originating from the surface accumulation of glycerol. However, the observed changes in the surface topography of the lyophilized foam did not affect its usable properties.

## 3. Materials and Methods

### 3.1. Chitosan

For the fabrication of the usable form of the topical haemostatic agents, chitosan ChitoClear hqg 95 (Primex ehf., Siglufjordur, Iceland) with an average molecular weight Mv = 373.0 kDa and a degree of deacetylation DD—81.0% was used.

### 3.2. Alginate

The second biopolymer applied for fabrication of usable forms of the topical haemostatic agents was sodium alginate Protanal LF 10/60 FT (FMC Biopolymer, Philadelphia, PA, USA) with a viscosity of 1% 20 °C, 34 mPas.

### 3.3. Supplementary Aids

Calcium chloride (POCh S.A., Avantor Performance Materials Poland S.A., Gliwice, Poland), sodium hydroxide (Sigma-Aldrich Co., St. Louis, MO, USA), lactic acid (Avantor Performance Materials Poland S.A., Gliwice, Poland), water for injection (Baxter Polska Sp. z o.o., Lublin, Poland), and glycerol (Fluka Co., St. Louis, MO, USA) were the supplementary aids for fabrication of the usable form of topical haemostatic agents.

The gauze selected for impregnation by the topical haemostatic agent was manufactured by Matopat, Toruń, Poland.

The usable forms of the topical haemostatic agent were packed in the medical grade Octasafe 70 foil (WIPAK, Nastola, Finland) and subjected to an irradiation sterilization.

### 3.4. Reference Topical Haemostatic Agents, Clinically Used

Celox™ (haemostatic granules; Medtrade Products Ltd., Crewe, UK) and Quikclot^®^ ACS (Z-Medica Corporation, Wallingford, CT, USA) topical haemostatic agents were used for the validation of the risk acceptance level.

### 3.5. Semi-Product—Powder

The chitosan/alginate micro- and nano-fibrids composition was used for the fabrication of the usable forms of the topical haemostatic agents, prepared according to [18,25,26].

The lyophilized powder made of an aqueous suspension of the chitosan/alginate micro- and nano-fibrids [18,27] was used as a reference.

### 3.6. Usable Forms of the Topical Haemostatic Agents

Two types of usable forms of the topical haemostatic agents including lyophilized foam and impregnated gauze-based on the composition of chitosan/alginate micro- and nano-fibrids were used in the study:

- Foam fabricated by the lyophilization of the complex chitosan/(calcium/sodium) alginate micro- and nano-fibrids mixture with a polymer content of 2.7 wt %. The plasticizer (glycerol) was added with the contribution of 0.7 parts per 1.0 by the weight of biopolymers. The process of freeze-drying was performed in the form sized 8 × 8 cm in the temperature from −35 °C to +10 °C, pressure range: 0.10 mbar–0.57 mbar for 24 h.
- Impregnated gauze with a length of 1 m received by the impregnation of the active layer of the chitosan/(calcium/sodium)alginate micro- and nano-fibrids with a polymer content of 1.3 wt %. The plasticizer (glycerol) was also added with the contribution of 0.6 parts per 1.0 by weight of biopolymers. The degree of impregnation amounted to 20.0 ± 0.5 g/m^2^. The impregnated gauze was freeze-dried at a temperature ranging from −35 °C to +10 °C, and a pressure range of 0.10 mbar–0.57 mbar for 24 h.

The drying process in both cases was performed by lyophilisation using a laboratory freeze-dryer Alpha 24 LSC (Martin Christ GmbH, Osterode am Harz, Germany).

All of the usable forms of the topical haemostatic agents were sterilized by irradiation (accelerated electrons) with a 25 kGy dose at the Institute of the Nuclear Chemistry and Technology (Warszawa, Poland).

### 3.7. Risk Analysis

The risk analysis was performed using the Failure Mode and Effects Analysis (FMEA) technique [18], taking into the account the guide given in EN ISO 14971:2012 and EN ISO 22442-1:2007 Standards, as described in [12,13]. During the test, the stability of the designed functionalities in usable forms of the tested topical haemostatic agents for the simulated one year was estimated.

### 3.8. Irradiation Sterilization

Irradiation sterilization by accelerated electrons was carried out at Institute of Nuclear Chemistry and Technology (Warszawa, Poland) using an Elektronika 10/10 accelerator (electron energy max. of 10 MeV and power of 15 kW, Institute of Nuclear Chemistry and Technology, Warszawa, Poland). Based on the guide described in EN ISO 11137-2:2013 [28], the irradiation dose of 25 kGy was established. The technical parameters of the irradiation process were as follows: transport speed of 0.380 m/min, initial current of 380 mA, and energy of 10 MeV.

### 3.9. Accelerated Ageing Process

The process of estimating the performance stability of the designed, usable form of the topical haemostatic agents was performed according to the guide given in ASTM F1980-07:2011 Standard, as described in [29]. For this study, a period of one year under real conditions (related to 52 days under accelerated ageing test conditions) was established.

The ageing test describes the transformed form of the Arrhenius equation:

(1) AAF=Q10⌈(TAA−TRT)10⌉

where: *AAF*—accelerated ageing factor; T_AA_—accelerated ageing temperature [°C]; T_RT_—storage temperature in real time of ageing of the sample [°C]; Q_10_—ageing factor based on the change kinetics of the selected property/material parameter at 10 °C temperature changes.

To calculate the actual ageing time, use the equation below:

(2) ATT=365AAF

where: *ATT*—time of accelerated ageing equivalent to the actual ageing time (1 year).

Assuming the parameter values, T_AA_ = 50 °C, T_RT_ = 22 °C, and Q_10_ = 2, the time of accelerated ageing is 52 days. Accelerated ageing was performed for samples after irradiation sterilization.

### 3.10. Fourier Transform Infrared Study (FTIR)

Samples (before and after irradiation sterilization) were characterized using a FTIR spectrophotometer (Thermo Scientific Nicolet iS10, Waltham, MA, USA) with KBr pellets. The spectra were obtained from 400–4000 cm^−1^ with a resolution of 4 cm^−1^. The background spectrum of the KBr pellet was subtracted from the FTIR spectra.

### 3.11. Scanning Electron Microscope Study (SEM)

SEM observations of the topography of the tested usable forms of the topical haemostatic agents were carried out using SEM Quanta 200 (FEI Co., Hillsboro, OR, USA).

### 3.12. Absorption under Free Soaking

The absorption under free soaking of the tested usable forms of the topical haemostatic agents was determined according to the guidelines described in EN 13726-1:2002/AC:2003 [30] Standard using the tested liquid containing NaCl and CaCl_2_·2H_2_O simulating the blood serum. The value of absorption under free soaking was defined as the average mass of the absorbed liquid per 1 g of the sample.

### 3.13. Moisture Vapour Transmission Rate (MVTR)

MVTR of the tested usable forms of the topical haemostatic agents in contact with water vapour was determined using the guidelines, as defined in EN 13726-2:2002 [31]. MVTR defines the permeability of the materials to allow water molecules to enter the outer atmosphere under certain conditions of humidity and temperature. During the test, the moisture vapour transmission through the dressings is measured as a mass difference. Liquid retention in the dressing can lead to severe skin damage. It is recommended that the dressing exhibit sufficient moisture vapour transmission capacity to prevent fluid accumulation under the dressing.

*MVTR* is calculated from the formula:

(3) $$MVTR=\left(W_{1}-W_{2}\right)\times 1000\times \frac{24}{T}$$

where *W*_1_ is the mass of the container (sample and water), *W*_2_ is the mass of the container (sample and water) after the test, and T is the test time measured in hours.

### 3.14. Surface Density

The surface density of the usable forms of the topical haemostatic agents was determined by the estimation of three measurements in width and length of the samples masses. The surface density was calculated according to following equation:

(4) $$Mp=\frac{m_{i}}{\left(d\times s\right)}\times 10^{6}$$

where *m_i_* is the mass of the sample [g]; *d* is the length of the sample, calculated as an average of three measurements [mm]; *s* is the width of the sample, calculated as an average of three measurements [mm].

## 4. Conclusions

Newly designed usable forms for topical haemostatic agents that were made from the suspension of the complex of micro- and nano-fibrids of chitosan/(calcium/sodium) alginate proved to be of high performance in the application of surgical interventions, medical first aid in massive bleeding, haemostasis emergency, bleeding resulting from multiple defects, combat injuries, and in patients with haemostasis problems.

The main innovative features of the haemostatic factor include the increase in the surface contact with morphotic blood constituent related to the activation of the haemostatic pathway; promoting the aggregation of the blood platelets due to a positive charge; and, improvement in the absorption capacity affecting the indirect concentration of the natural haemostatic factors that promote natural activation of the haemostatic pathway.

The risk analysis remarkably supported the process of defining the usable properties, as well as appointed the process of verifying the performance of the topical haemostatic agents depending on their usable forms and potential scope of the clinical application, such as surgical interventions, medical first aid in case of massive bleeding, haemostasis emergency, bleeding resulting from multiple defects, combat injuries, and in patients with haemostasis problems. The above statements have been verified during the present study. A proper application of the risk analysis will support the optimal selection of the design of the topical haemostatic agents being the process innovations.

Risk management is a main tool useful for the optimal design of the research and development programme. It rationally optimizes the R&D pathway by indicating the most appropriate verification tests to confirm the assumed thesis of the research. The paper shows idea of the adaptation of the risk management in the research as a tools helping scientist at screening the most suitable testing methods in complexing way the scientific thesis. In reference to the above, the risk management as an element of design thinking is useful tool for scientists’ design at the optimal research programme, which verified the assumed thesis. Thus, it has significant potentiality for the implementation in the research & development phase not only from companies’ point of view due but also in an aspect of the quality of the fundamental research realization. A comparison of the usable properties of the developed usable forms with the commercially available haemostatic dressings showed the supremacy of the former. Both usable forms are characterized by high absorption under free soaking that indirectly supports the process of the haemostasis and MVTR. Moreover, the accelerated ageing study allows for estimating the performance stability of the developed topical agents during the storing and transportation.

A validation of the thesis verified during the present study will be performed on a more complex and wider scale during the biocompatibility and clinical studies.
